# The effects of Co on the enhancement of magnetic properties by modifying the intergranular phase in Nd-Fe-B alloys

**DOI:** 10.1038/s41598-018-36583-x

**Published:** 2019-02-11

**Authors:** Y. Liang, Q. Deng, X. H. Tan, H. Li, H. Xu

**Affiliations:** School of Materials Science and Engineering, Shanghai University, Shanghai, 200444 China

## Abstract

In Nd_2_Fe_14_B-based permanent materials, the intergranular phase has a strong influence on magnetic properties. Here, we study the effect of partial substitution of Fe by Co on the microstructure to gain insight into the mechanism of enhancing magnetic properties of (Nd_0.8_Pr_0.2_)_2.2_Fe_14−x_Co_x_B (x = 0, 1.75, 2, 2.25) alloys. Our results show that the substitution Co for Fe changes the magnetic properties obviously by tuning the chemistry and distribution of the intergranular phase between hard magnetic grains. In particular, for (Nd_0.8_Pr_0.2_)_2.2_Fe_12_Co_2_B (x = 2) alloy, no obvious intergranular phase is observed. And the through-thickness homogeneity and ultrafine microstructure with an average size of ~25 nm is obtained, which produces maximum product ((BH)_max_) of 141 kJ/m^3^, 29% higher than that of quaternary alloy. Our findings provide a new idea to design prospective permanent alloys with increased magnetic properties by tuning the distribution and chemical composition of the intergranular phase.

## Introduction

Nd_2_Fe_14_B-typed permanent magnets (PMs) have found applications in a wide range of fields including electronic, data processing and medical devices due to their outstanding hard magnetic properties^1,2^. It is known that rapid solidification by melt spinning is one of the most important methods to produce melt-spun ribbons consisting of randomly oriented Nd_2_Fe_14_B grains with an average diameter of less than 100 nm, which are currently used as raw materials for the production of bonded magnets and hot-pressed magnets^3^. Therefore, optimizing the microstructure of melt-spun ribbons is critical to improving the magnetic properties of Nd-Fe-B PMs. Moreover, in recent years, there has been great effort in developing economically more attractive permanent materials because of the price volatility and limited availability of critical rare earth metals (RE), such as Dy and Nd^4^. For example, Pathak *et al*. reported an unexpected increase of the intrinsic coercivity (H_c_^i^ = 17.7 kOe) of Ce and Co co-doped (Nd_0.8_Ce_0.2_)_2.4_Fe_12_Co_2_B melt-spun ribbons at room temperature, which was larger than that of 5.9 wt% Dy containing ribbons^5^. The segregation of heavy elements at the intergranular phase was speculated to play an important role on the improvement of the coercivity, however, the mechanism of enhancing the coercivity is still veiled.

It has been found that the composition and distribution of intergranular phase between Nd_2_Fe_14_B (2:14:1) grains have a strong influence on the coercivity of Nd_2_Fe_14_B-based PMs^6^. Two different types of intergranular phase are observed: i) non-magnetic intergranular phase with large amount of RE (>65 at.%) which improves the coercivity by promoting a magnetic isolation of 2:14:1 grains^7^; and ii) ferromagnetic intergranular phase containing large fractions of Fe and/or Co more than 65 at.% leads to an improvement of remanence by enhancing the inter-grain coupling^8^. It is worth noting that the role of Co distribution in both types of intergranular phase is under debate. For non-magnetic intergranular phase of hot-deformed Nd-Fe-B magnets, substitution of Co for Fe led to the formation of a Co-enriched layer at the edges of 2:14:1 grains resulting in a reduction of the magnetocrystalline anisotropy of 2:14:1 phase^9^, whereas the segregation of Co was found at the interface between the intergranular phase and 2:14:1 phase acting as nucleation sites for reversed magnetic domains^10^. On the other hand, the enrichment of Co was observed at the magnetic intergranular phase and further promoted by magnetic field annealing, which resulted in an improvement of remanence by strengthening the exchange coupling interaction between hard magnetic grains of Nd-Ce-Fe-Co-B alloys^11^. Hence, the effect of Co addition on the coercivity needs to be further investigated.

Remanence is another extrinsic magnetic property and it is intimately related to the microstructure of a magnetic material. One effective way of increasing the remanence is to refine the grain size by improving the inter-grain exchange interaction between 2:14:1 grains^12^. However, achievement of through-thickness homogeneity with an average size less than 50 nm in the ribbon microstructure during melt-spinning is extremely difficult due to some major factors including fabrication conditions and composition of alloys. Our previous work found homogenous microstructure with an average size less than 40 nm through-thickness Ce-Fe-B melt-spun ribbons was obtained by optimizing fabrication parameters (e.g. chamber pressure and wheel speed) during melt-spinning^13^. Therefore, a refiner microstructure is expected by further adjusting the composition of alloys. Atom probe tomography (APT) can determine quantitatively the local composition distributions in three dimensions with atom resolution and has been used widely to illustrate the distribution of additive element^14,15^, microstructural evolution^16,17^ and the role of intergranular phase^18,19^ in magnetic ribbons. For example, Cu and Nb addition is reported to decrease the grain size effectively resulting in an improvement of magnetic properties of nanocomposite alloys^15^. It is found by APT technique that Y-containing intergranular phase contributes to the enhanced domain wall pinning at the grain boundary in (Nd_0.5_Y_0.25_Dy_0.25_)_1.8_Zr_0.4_Co_1.5_Fe_12.5_B ribbons^19^. In this study, we investigated the microstructure and magnetic properties of (Nd_0.8_Pr_0.2_)_2.2_Fe_14−x_Co_x_B (x = 0, 1.75, 2, 2.25) melt-spun ribbons to understand how Co-substitution for Fe could be employed to tailor the magnetic properties by tuning the distribution, type and chemistry of the intergranular phase. Partial substitution of Nd by Pr is chosen because Pr addition can promote uniform distribution of intergranular phase resulting in an increase of magnetic properties in Nd-Fe-B alloys^20,21^. The results show that the substitution Co for Fe affects the type, chemistry, and distribution of the intergranular phase. For x = 2 alloy, no obvious intergranular phase is observed, and the through-thickness homogeneity and ultrafine microstructure with an average size of ~25 nm is obtained. It produces maximum product ((BH)_max_) of 141 kJ/m^3^, which is 29% higher than that of quaternary alloy.

## Results

**Figure 1:**
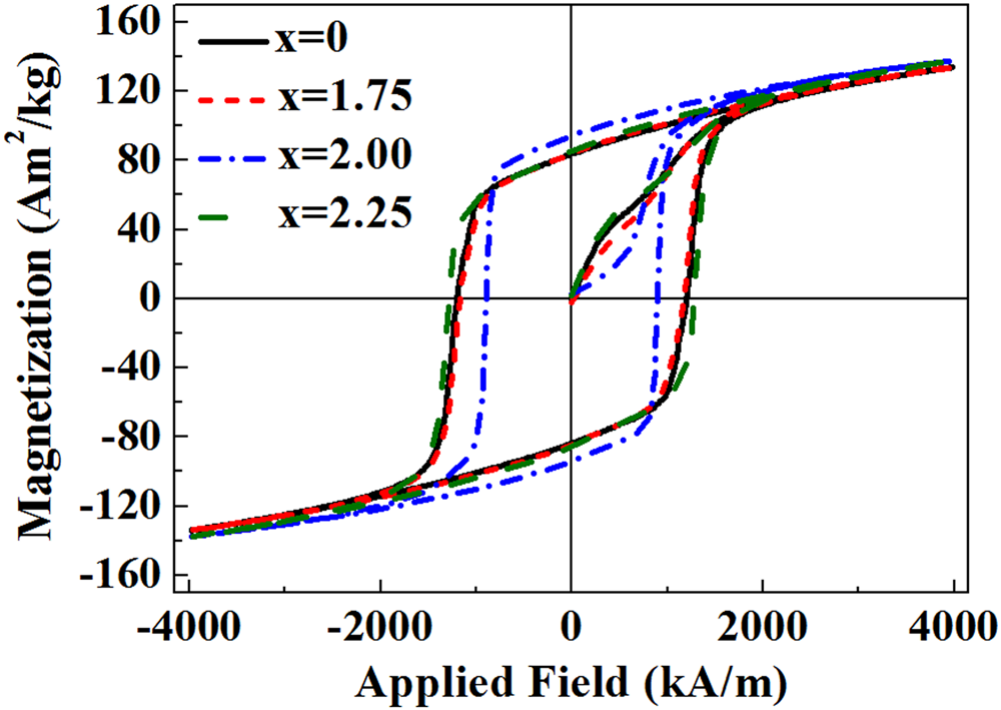
Hysteresis loops of (Nd_0.8_Pr_0.2_)_2.2_Fe_14−x_Co_x_B (x = 0, 1.75, 2, 2.25) melt-spun ribbons at room temperature.

Figure 1 shows the hysteresis loops of (Nd_0.8_Pr_0.2_)_2.2_Fe_14−x_Co_x_B (x = 0, 1.75, 2, 2.25) melt-spun ribbons. The key magnetic property parameters are shown in Table 1. The intrinsic coercivity (H_c_^i^) decreases, reaching a minimum at x = 2, and then increases at x = 2.25. In contrast, the remanence (B_r_) and (BH)_max_ increase, reaching a maximum at x = 2 with a value of 0.91 T and 141 kJ/m^3^, and then decrease with further Co addition.

**Table 1:** The intrinsic coercivity, *H*_*c*_^*i*^, the remanence, *B*_*r*_, the maximum product, *(BH)*_*max*_, and the average grain size of wheel surface and free surface of (Nd_0.8_Pr_0.2_)_2.2_Fe_14−x_Co_x_B (x = 0, 1.75, 2, 2.25) melt-spun ribbons.

Co content	*H*_*c*_^*i*^ *(kA/m)*	*B*_*r*_ *(T)*	*(BH)*_*max*_ *(kJ/m* ^*3*^ *)*	*Average grain size (nm)*	
				wheel surface	free surface
x = 0	1200	0.80	109	43 ± 4	71 ± 2
x = 1.75	1172	0.81	112	56 ± 1	74 ± 2
x = 2	896	0.91	141	25 ± 1	28 ± 1
x = 2.25	1280	0.83	113	58 ± 1	71 ± 2

**Figure 2:**
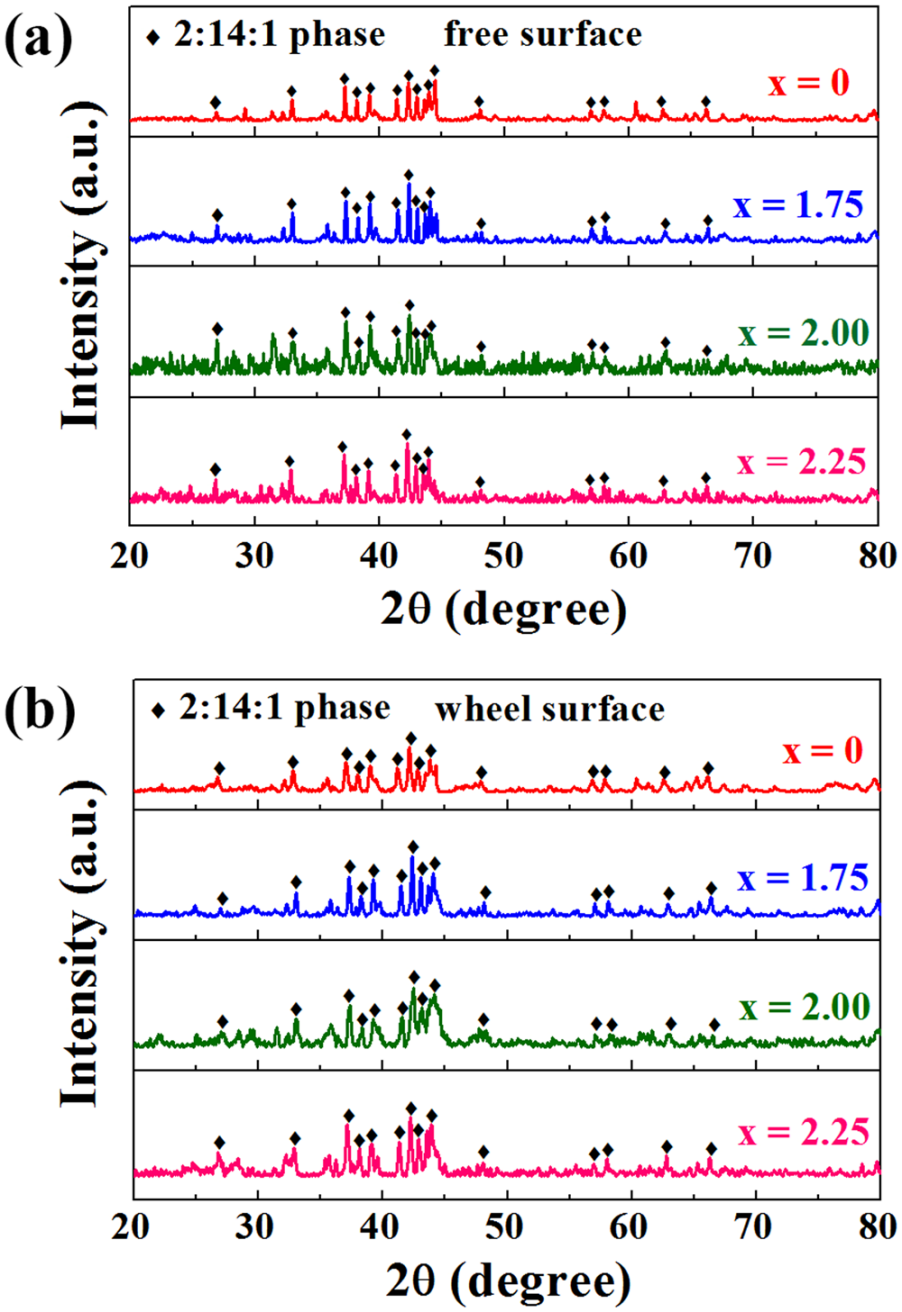
XRD patterns of the free surface (**a**) and wheel surface (**b**) of (Nd_0.8_Pr_0.2_)_2.2_Fe_14−x_Co_x_B (x = 0, 1.75, 2, 2.25) melt-spun ribbons.

The XRD patterns of the free surface and wheel surface (the surface in contact with the copper wheel) of (Nd_0.8_Pr_0.2_)_2.2_Fe_14−x_Co_x_B (x = 0, 1.75, 2, 2.25) melt-spun ribbons is shown in Fig. 2. A single 2:14:1 phase is observed on both surfaces for all samples indicating that Co addition does not change the phase constitution. The average grain size is calculated by Scherrer formula^22^ and given in Table 1. It is found that the wheel surface of ribbons has finer grains than the free surface in all samples. For x = 0, 1.75, and 2.25 alloys, the average size of wheel surface increases from 43 ± 4 nm to 58 ± 1 nm, while the average size of free surface changes marginally (≈71 nm). In particular, for x = 2 alloy, the average grain size is 25 ± 1 nm and 28 ± 1 nm close to the wheel surface and free surface, respectively. It indicates that proper amount of Co addition (x = 2) decreases effectively the grain size and refines the microstructure.

**Figure 3:**
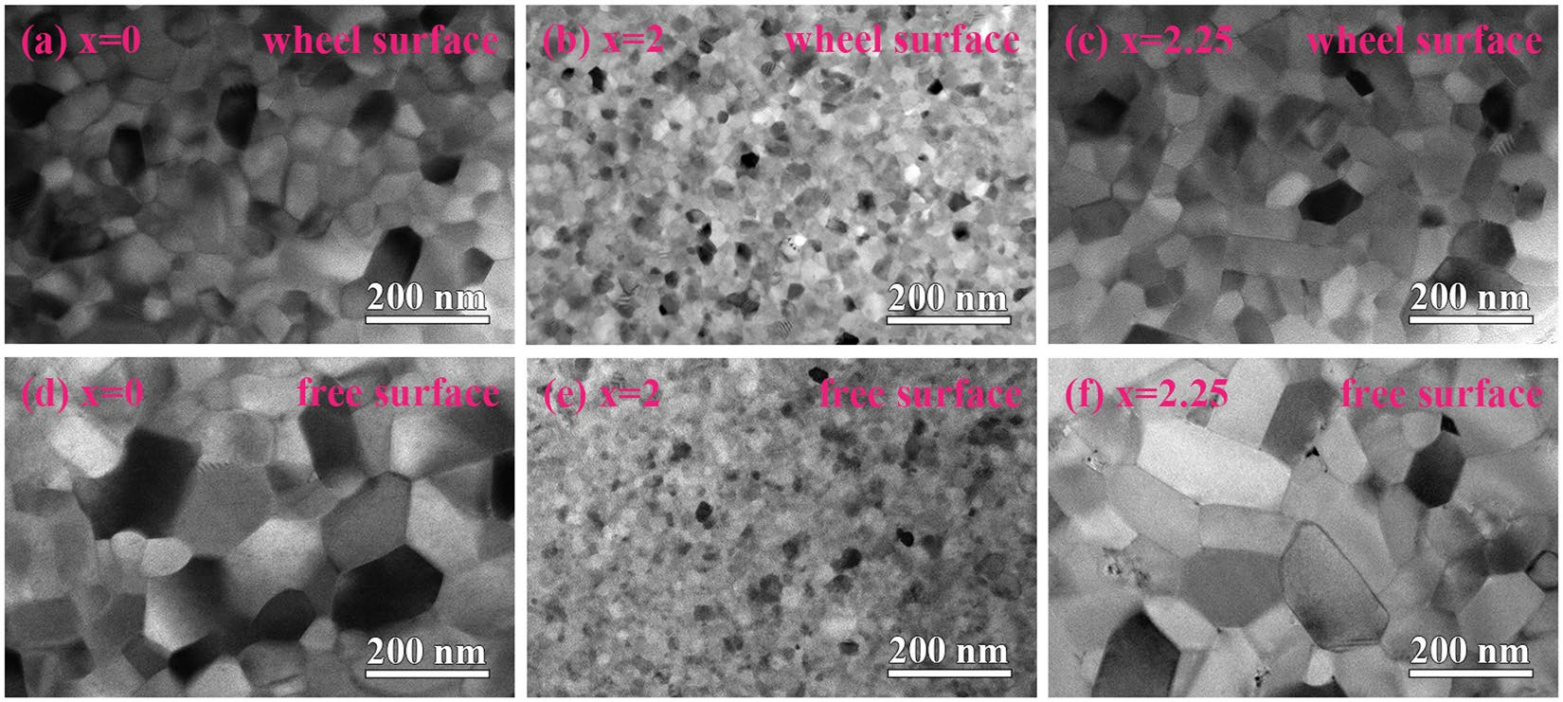
Transmission electron microscopy (TEM) images of cross-sectional region near the wheel surface and free surface of (Nd_0.8_Pr_0.2_)_2.2_Fe_14−x_Co_x_B melt-spun ribbons; (**a**,**d**) x = 0, (**b**,**e**) x = 2, and (**c**,**f**) x = 2.25.

Figure 3a–f show TEM result of cross-sectional region near the wheel surface and free surface of (Nd_0.8_Pr_0.2_)_2.2_Fe_14−x_Co_x_B (x = 0, 2, 2.25) melt-spun ribbons, respectively. It is shown that a homogeneous microstructure through the thickness is observed in all samples. The grain size distribution was determined from the TEM images, see Supplementary Fig. S1. The uniform and finer microstructure with an average size of 25 ± 4 nm is obtained through the thickness of ribbon by adjusting Co addition for x = 2. It is consistent with values calculated by Scherrer formula using XRD data. That is, the through-thickness homogeneity with an average size less than 30 nm in the ribbon microstructure during melt-spinning is obtained for (Nd_0.8_Pr_0.2_)_2.2_Fe_12_Co_2_B (x = 2) alloy.

**Figure 4:**
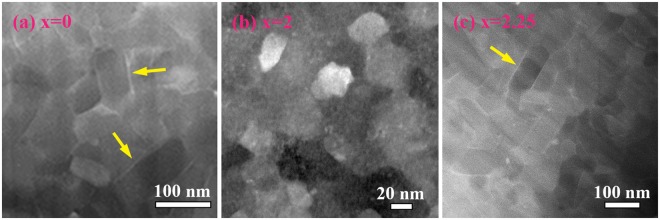
HAADF images of cross-sectional region near the wheel surface of (Nd_0.8_Pr_0.2_)_2.2_Fe_14−x_Co_x_B melt-spun ribbons. (**a**) x = 0, (**b**) x = 2, and (**c**) x = 2.25.

For further investigation of the microstructure and analysis of chemical composition of the intergranular phase, the wheel surface of ribbons was chosen. For quaternary (Nd_0.8_Pr_0.2_)_2.2_Fe_14_B alloy, the high-angle-annular-dark-field (HAADF) images show that 2:14:1 grains are surrounded by thin intergranular layers with bright contrast along the grain boundaries (GBs), marked as arrows (see Fig. 4a). However, no bright contrast is observed in (Nd_0.8_Pr_0.2_)_2.2_Fe_12_Co_2_B (x = 2) alloy indicating no segregation of heavy elements at GBs (see Fig. 4b). With further increasing Co content (x = 2.25), the intergranular phase is observed, see an arrow in Fig. 4c. It suggests that Co addition can modify the distribution of the intergranular phase.

**Figure 5:**
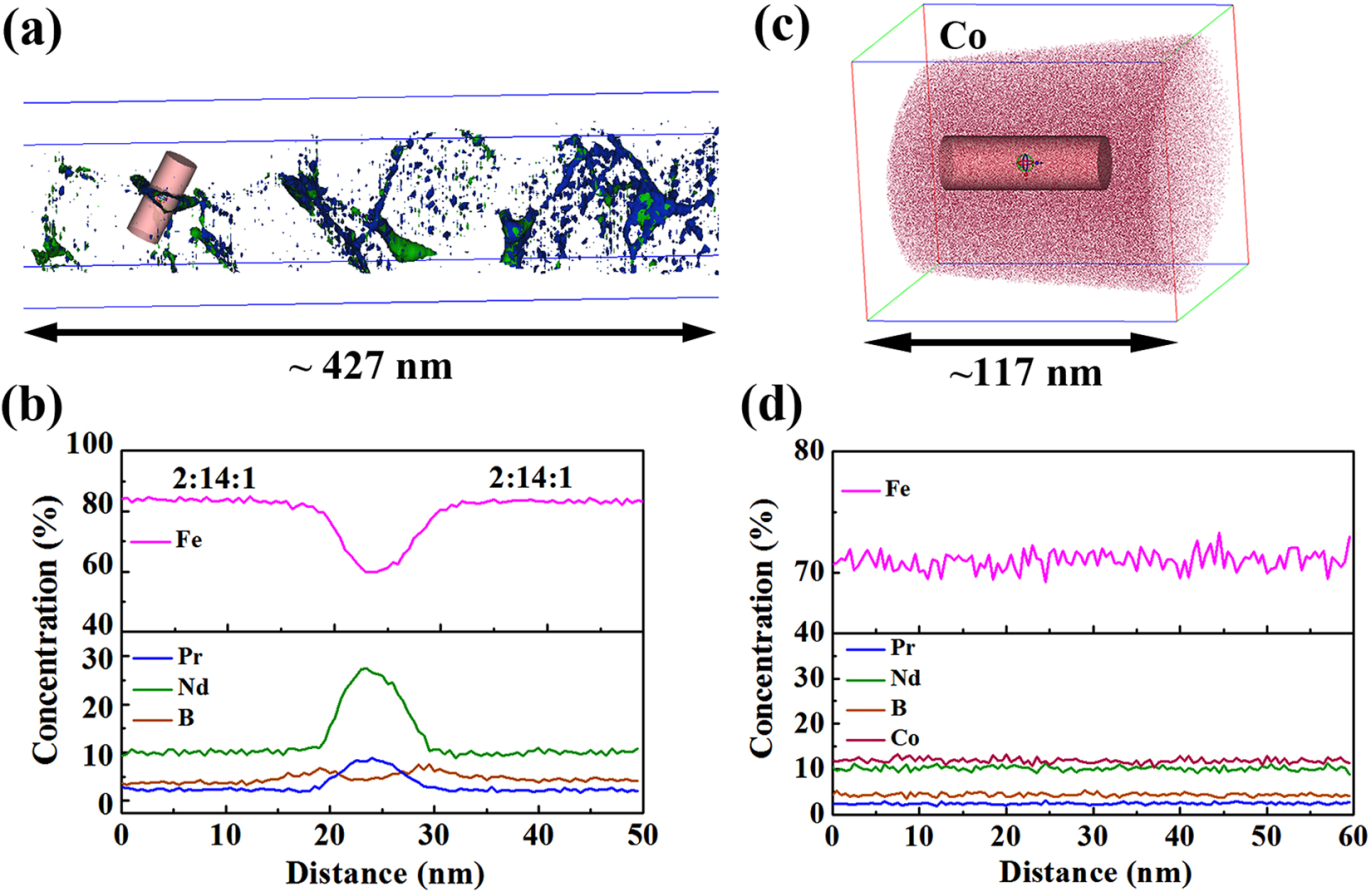
(**a**) Atom probe tomography (APT) reconstruction of (Nd_0.8_Pr_0.2_)_2.2_Fe_14_B melt-spun ribbons (x = 0) using 4 at.% Pr (blue color) and 14 at.% Nd isoconcentration surfaces (green color); (**b**) the corresponding concentration profiles obtained from the selected volume with a size of 10 nm × 10 nm × 50 nm in (**a**) for x = 0 alloy; (**c**) APT reconstruction of (Nd_0.8_Pr_0.2_)_2.2_Fe_12_Co_2_B (x = 2) alloy; (**d**) the corresponding concentration profiles obtained from the selected volume with a size of 20 nm × 20 nm × 60 nm in (**c**) for x = 2 alloy.

The atom probe tomography (APT) reconstruction of (Nd_0.8_Pr_0.2_)_2.2_Fe_14_B (x = 0) alloy is shown in Fig. 5a. An isoconcentration surface of 4 at.% Pr (blue color) and 14 at.% Nd (green color) were used to visualize and identify the intergranular phase. It is shown clearly that Nd and Pr are enriched at the intergranular phase. An analyzed volume of 10 nm × 10 nm × 50 nm in Fig. 5a is selected, and the corresponding concentration depth profiles show that the average amount of Nd and Pr at the intergranular phase is 19 ± 1 at.% and 6 ± 1 at.%, respectively (see Fig. 5b). In addition, a segregation of B is found at the surface between the intergranular phase and 2:14:1 phase, i.e., 7 ± 1 at.% B at the interface, higher than 4 ± 1 at.% B at the intergranular phase. It is noted that the average concentration of Fe (70 ± 1 at.%) is found at the intergranular phase, which is higher than 65 at.% reported by Sepehri-Amin^8^ indicating that the intergranular phase is ferromagnetic. Figure 5c shows APT reconstruction of Co element in (Nd_0.8_Pr_0.2_)_2.2_Fe_12_Co_2_B (x = 2) alloy. No segregation of elements is observed at GBs. Figure 5d is the concentration depth profile from the analysis volume of 20 nm × 20 nm × 60 nm in Fig. 5c. All elements have uniform distribution suggesting that proper amount of Co substitution for Fe can eliminate the segregation of elements along the GBs.

**Figure 6:**
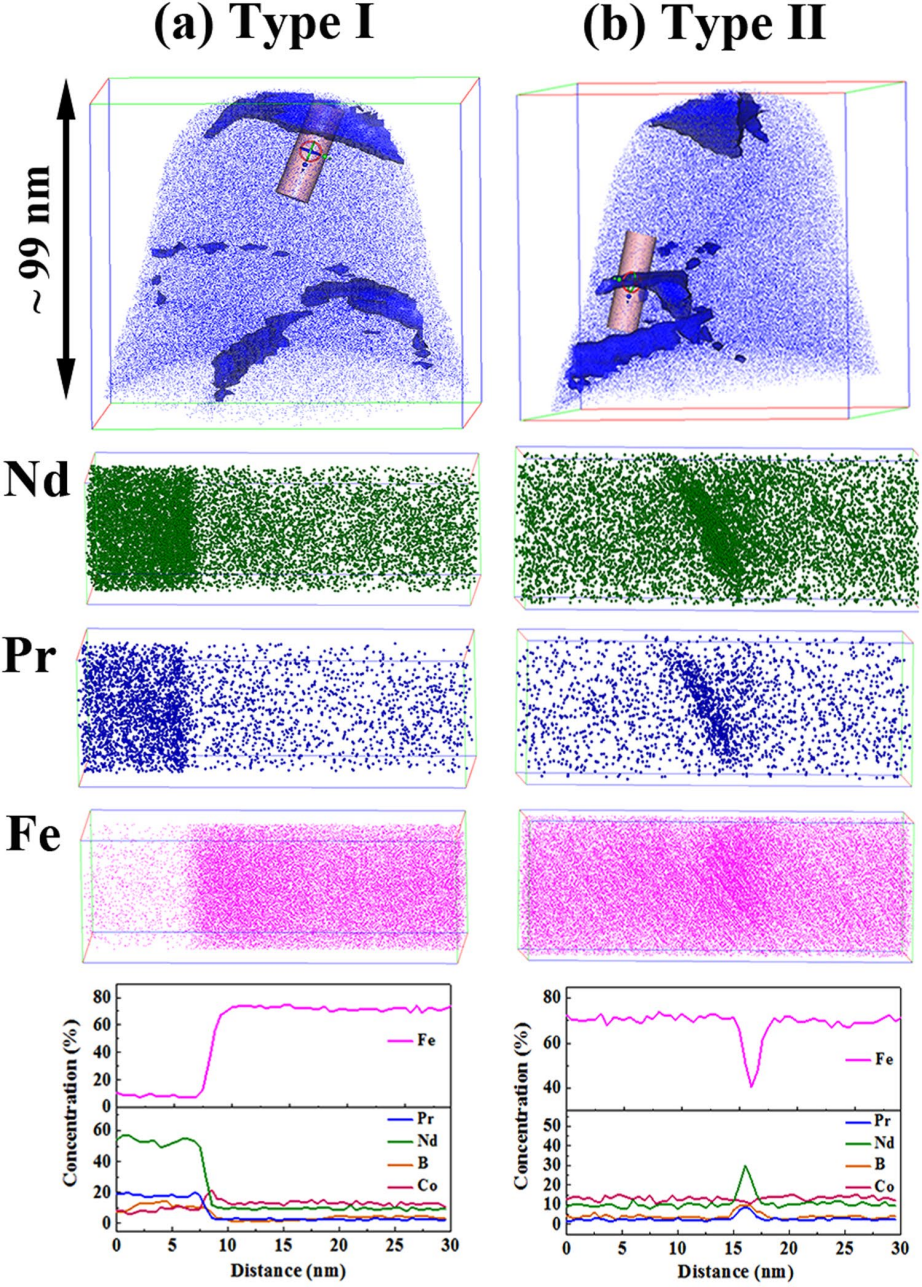
APT result of (Nd_0.8_Pr_0.2_)_2.2_Fe_11.75_Co_2.25_B (x = 2.25) alloy. (**a**) type I intergranular phase; (**b**) type II intergranular phase.

With further increasing Co addition for x = 2.25 alloy, however, Fig. 6 shows two types of intergranular phase identified by the isoconcentration surface of 4 at.% Pr (blue color). The APT maps of Nd, Pr and Fe within the cylinder volume with a size of 10 nm × 10 nm × 30 nm in which the type I intergranular phase is distinguished with the segregation of Nd and Pr, and depletion from Fe (see Fig. 6a). The corresponding concentration depth profiles show that type I intergranular phase contains about 52 ± 1 at.% Nd, 18 ± 1 at.% Pr, 10 ± 1 at.% Fe and 10 ± 1 at.% Co. That is, the type I intergranular phase contains large amount of RE (Nd + Pr ≈ 70 at.%) indicating type I intergranular phase is non-ferromagnetic. Furthermore, Co is enriched at the interface between the intergranular phase and 2:14:1 phase, i.e., 20 ± 1 at.% Co at the interface, higher than 14 ± 1 at.% Co in the 2:14:1 phase. Figure 6b shows the segregation of Nd and Pr in type II intergranular phase, in which the concentration depth profiles calculated from the cylinder volume exhibit that the amount of Fe + Co is about 67 ± 1 at.% suggesting this type of intergranular phase is ferromagnetic. The enrichment of Co at the interface between the intergranular phase and 2:14:1 phase is not observed in type II intergranualr phase. Moreover, Co addition changes the enrichment of B from the interface between the intergranular phase and the 2:14:1 phase for x = 0 alloy to the segregation of B atoms at the intergranular phase for x = 2.25 alloy.

## Discussion

The microstructure analyses of the (Nd_0.8_Pr_0.2_)_2.2_Fe_14_B (x = 0) alloy show that 2:14:1 grains are surrounded by intergranular phase enriched Nd and Pr. A segregation of B is found at the surface between the intergranular phase and 2:14:1 phase. Furthermore, large amount of ferromagnetic element Fe (≈70 at.%) is observed at the intergranular phase, higher than 65 at.% reported by Sepehri-Amin^8^, and is considered to be ferromagnetic. However, with Co addition for (Nd_0.8_Pr_0.2_)_2.2_Fe_11.75_Co_2.25_B (x = 2.25) alloy, besides ferromagnetic intergranular phase, another type of non-ferromagnetic intergranular phase is found. The segregation of Co at the interface between the intergranular phase and 2:14:1 phase is observed. Moreover, Co addition promotes the enrichment of B atoms at the intergranular phase. That is, the enrichment of non-ferromagnetic elements, such as Pr, Nd and B, is found at the intergranular phase, which is helpful to increase the covercivity by weakening the inter-grain coupling of 2:14:1 grains. Therefore, we consider that both increase of H_c_^i^ (from 1200 kA/m for x = 0 alloy to 1280 kA/m for x = 2.25 alloy) and B_r_ (from 0.80 T for x = 0 alloy to 0.83 T for x = 2.25 alloy) is due to the role of competition between two types of intergranular phase on the magnetic properties. It is worth noting that no obvious intergranular phase is observed for (Nd_0.8_Pr_0.2_)_2.2_Fe_12_Co_2_B (x = 2) alloy. Intergranular phase was reported to retard grain growth resulting in the microstructural refinement^23^. In our work, however, HAADF image (see Fig. 4b) shows that uniform and fine microstructure with an average size of about 25 nm without intergranular phase is observed in (Nd_0.8_Pr_0.2_)_2.2_Fe_12_Co_2_B (x = 2) melt-spun ribbons.

**Figure 7:**
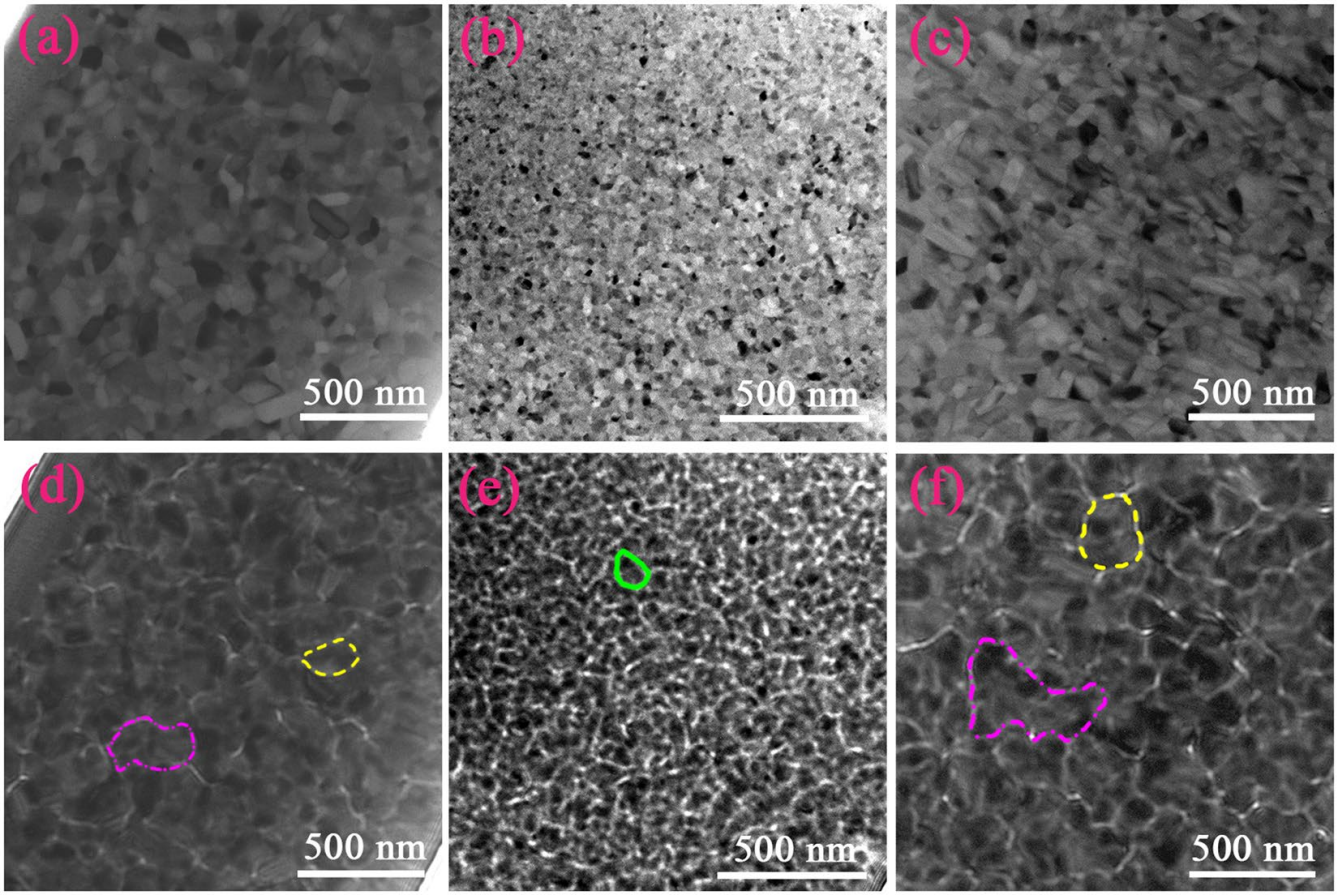
TEM bright field images and Lorentz microscope images of (Nd_0.8_Pr_0.2_)_2.2_Fe_14−x_Co_x_B melt-spun ribbons; (**a**,**d**) x = 0, (**b**,**e**) x = 2, and (**c**,**f**) x = 2.25.

Figure 7a–c and d–f show Fresnel images of a region near the wheel surface of (Nd_0.8_Pr_0.2_)_2.2_Fe_14−x_Co_x_B (x = 0, 2, 2.25) melt-spun ribbons in mode of just-focus and under-focus, respectively. All images were observed in the demagnetization state. Big domain size (marked in pink dash) and small domain size (marked in yellow short dash) are both observed in x = 0 alloy (see Fig. 7d) and in x = 2.25 alloy (see Fig. 7f). However, similar domain size (marked with a solid line circle in Fig. 7e) is observed in x = 2 alloy. The average domain size is 223 ± 35 nm, 90 ± 5 nm and 263 ± 34 nm in width for x = 0, 2, and 2.25, respectively. It is noted that the average domain size is much larger than the average grain size of 43 ± 4 nm, 25 ± 1 nm and 58 ± 1 nm. That is, a domain includes several grains, which is termed as an interaction domain^24^. It indicates the presence of strong exchange coupling interaction between neighboring 2:14:1 grains. It is due to the existence of ferromagnetic intergranular phase in x = 0 and x = 2.25 alloys and the direct contact of 2:14:1 grains in x = 2 alloy. The variation of interaction domain size is attributed to the various grain morphology and non-uniform grain size^25^.

**Figure 8:**
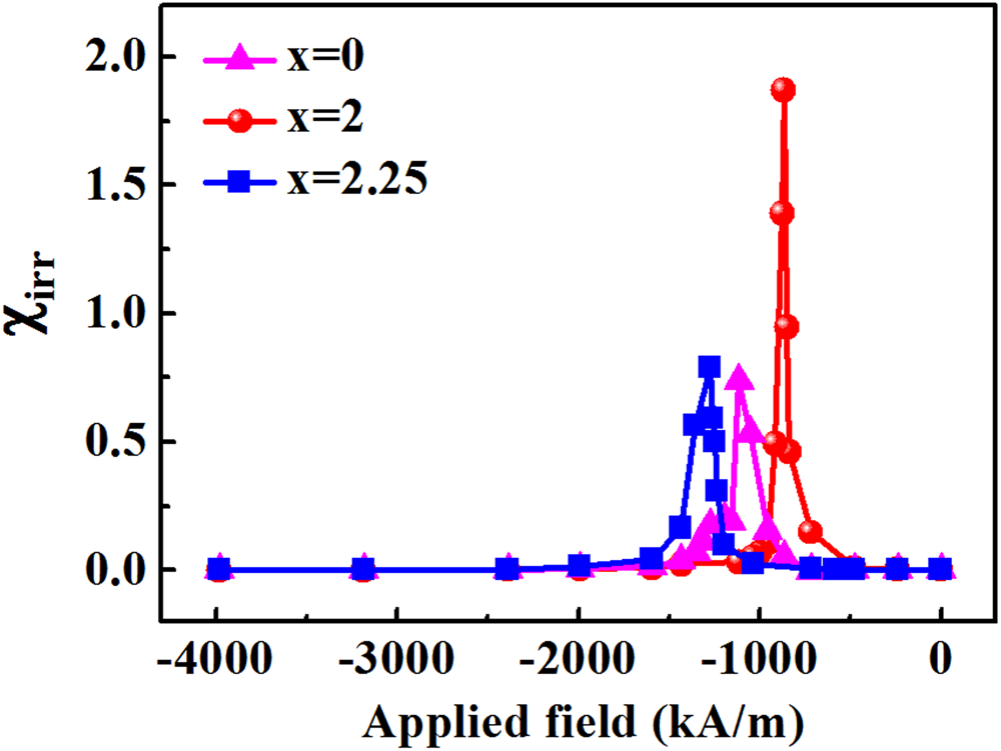
The irreversible susceptibility, *χ*_*irr*_, curves as a function of applied field for (Nd_0.8_Pr_0.2_)_2.2_Fe_14−x_Co_x_B (x = 0, 2, 2.25) melt-spun ribbons.

An effective method of understanding the exchange coupling interaction is via irreversible magnetic susceptibility (*χ*_*irr*_) plot^26^. Here, *χ*_*irr*_ = d*M*_irr_ */*d*H*, *M*_irr_ is the irreversible magnetization and can be obtained by a dc demagnetization (DCD) experiment as described in ref.^27^. Figure 8 shows *χ*_*irr*_ curves for (Nd_0.8_Pr_0.2_)_2.2_Fe_14−x_Co_x_B (x = 0, 2, 2.25) melt-spun ribbons. The narrow and intensive peak in *χ*_irr_ curve indicates that each grain couples well with its neighboring grains due to exchange coupling between magnetic phases. The (Nd_0.8_Pr_0.2_)_2.2_Fe_12_Co_2_B (x = 2) alloy shows a narrower and higher peak than x = 0 and x = 2.25 alloys suggesting a stronger exchange coupling interaction. Hence, the remanence is enhanced by about 14% from 0.80 T for x = 0 alloy to 0.91 T for x = 2 alloy.

During rapid solidification, achievement of through-thickness homogeneity in the ribbon microstructure appears to be limited by unidirectional heat flow through the solidified portion of the ribbon to the copper wheel^28^. Some factors influencing heat transfer in rapid solidification include wheel speed, mould surface, melt atmosphere, and melt composition^29^. In our work, the wheel speed, mould material, and melt atmosphere is 15 m/s, copper wheel, and argon atmosphere, respectively, and are not changed during melt-spinning. Thus, melt composition plays a significant role on the heat transfer and determines the final microstructure of melt-spun ribbons. For the (Nd_0.8_Pr_0.2_)_2.2_Fe_14_B (x = 0) alloy, the intergranular phase enriched Nd and Pr between 2:14:1 grains are observed (see Fig. 5a). It is due to the rejection of the excess Nd and Pr elements with respect to the stoichiometry composition of the Nd_2_Fe_14_B phase. However, the disappearance of intergranular phase of Co addition (x = 2) alloy may due to an enlargement of the undercooling resulting in the increase of the nucleation rate and suppression of the formation of intergranular phase between 2:14:1 grains. With further Co addition (x = 2.25), ferromagnetic and non-ferromagnetic intergranular phases are both observed indicating that increasing Co content stimulates the segregation of Nd and Pr at the intergranular phase.

From the sound fundamental understanding about heterogeneous nucleation, the magnitude of the heterogeneous nucleation barrier is given by^29^,

1 $${{{\rm{\Delta }}{\rm{G}}}^{\ast }}_{{\rm{het}}}={{{\rm{\Delta }}{\rm{G}}}^{\ast }}_{{\rm{\hom }}}\times f(\theta )$$

where ΔG^*^_hom_ is the magnitude of the homogeneous nucleation barrier, and *θ* is the wetting angle.

That is, the activation barrier for nucleation is dependent on *θ*. Our results show that proper amount of Co addition (x = 2) decreases effectively the grain size suggesting Co addition may reduce *θ* by enhancing the wettability of liquid metal with the mould surface, which increases the nucleation rate and refines the grain size due to reducing the energy barrier to nucleation. Hence, the ultrafine microstructure with an average size of ~25 nm is obtained, which produces (BH)_max_ of 141 kJ/m^3^, 29% higher than 101 kJ/m^3^ of quaternary alloy, and 41% higher than 100 kJ/m^3^ of (Nd_0.8_Ce_0.2_)_2.4_Fe_12_Co_2_B ribbons reported in ref.^5^. It is worth mentioning that the average Nd_2_Fe_14_B grain diameter with 23 nm was observed in Fe_82_Nd_12_B_6_ ribbons^30^. The remanence with 1.02 T is higher than 0.91 T of x = 2 ribbons, which may ascribe to stronger exchange coupling interaction between Nd_2_Fe_14_B grains. Moreover, it is reported that the coercivity with 2021.8 kA/m and (BH)_max_ with 156.2 kJ/m^3^ is obtained in decoupled Pr_15_Fe_78_B_7_ ribbons and stoichiometric Pr_12_Fe_82_B_6_ ribbons, respectively^31^. The values of coercivity and (BH)_max_ are both higher than those of our work, which is due to larger anisotropy field of Pr_2_Fe_14_B (75 kOe) than 73 kOe of Nd_2_Fe_14_B^3^.

In conclusion, the microstructure and magnetic properties of (Nd_0.8_Pr_0.2_)_2.2_Fe_14−x_Co_x_B (x = 0, 1.75, 2, 2.25) alloys are investigated. The results show that the substitution Co for Fe can adjust the distribution, type and chemistry of the intergranular phase. Ferromagnetic intergranular phase is found in (Nd_0.8_Pr_0.2_)_2.2_Fe_14_B (x = 0) alloy, whereas non-ferromagnetic and ferromagnetic intergranular phase are both observed for Co-containing with x = 2.25 alloy. In comparison to the (Nd_0.8_Pr_0.2_)_2.2_Fe_14_B (x = 0) alloy, both increase of H_c_^i^ and B_r_ of (Nd_0.8_Pr_0.2_)_2.2_Fe_11.75_Co_2.25_B (x = 2.25) alloy is because of the role of competition between two types of intergranular phase on the magnetic properties. For x = 2 alloy, no obvious intergranular phase is observed, and the ultrafine microstructure with an average size of ~25 nm through-thickness homogeneity is obtained. It improves the remanence significantly resulting in an achievement of (BH)_max_ with 141 kJ/m^3^, 29% higher than that of quaternary alloy without Co addition, and 41% higher than that of (Nd_0.8_Ce_0.2_)_2.4_Fe_12_Co_2_B ribbons reported in ref.^5^.

## Methods

Ingots of nominal composition (Nd_0.8_Pr_0.2_)_2.2_Fe_14−x_Co_x_B (x = 0, 1.75, 2, 2.25) were prepared by arc melting the mixture of pure metals Nd (99.5%), Pr (99.5%), Fe (99.5%), Co (99.999%), and a FeB pre-alloy in an argon atmosphere. Each ingot was re-melted four times for homogenization. Ribbons were produced by melt-spinning in an argon atmosphere at a wheel speed of 15 m/s. During melt-spinning, the quenching temperature and chamber pressure were controlled at 1588 K ± 5 K and 0.05 MPa. The nozzle at the bottom of a quartz tube was positioned 8 mm above the copper wheel surface. Magnetic properties were measured using a Quantum Design physical property measurement system (PPMS) (Quantum Design, San Diego, CA, USA) with a maximum magnetic field of 5 T. X-ray diffraction (XRD) patterns were collected using a D/max-2550 diffractometer with Cu K*α* radiation (Rigaku Corporation, Akishima-Shi, Tokyo, Japan). Cross-section samples near the wheel surface and free surface of ribbons for transmission electron microscopy (TEM), and near wheel surface for atom probe tomography (APT) observations were made by a Helios 600i focus ion beam (FIB) system (FEI Corporate, OR, USA), as shown in Supplementary Figure S2. The bright field image and high-angle-annular-dark-field (HAADF) image were carried out by a JEM-2100F scanning transmission electron microscope (STEM) (JEOL Ltd., Akishima, Tokyo, Japan). Lorentz microscope images were obtained using the JEM-2100F in Fresnel mode (JEOL Ltd., Akishima, Tokyo, Japan). The APT characterizations were performed in ultra-high vacuum (<10^−8^ Pa) on a CAMECA Instruments LEAP_4000X HR local electrode atom probe (Ametek Inc, Berwyn, PA, USA). The specimens were analyzed in voltage mode with a specimen temperature at 50 K with a target evaporation rate of 1%, and the energy of pulse laser is 60 pJ.

## Electronic supplementary material

The effects of Co on the enhancement of magnetic properties by modifying the intergranular phase in Nd-Fe-B alloys
