# Supplementary material for: The effects of Co on the enhancement of magnetic properties by modifying the intergranular phase in Nd-Fe-B alloys
Source: Sci Rep. 2019 Feb 11;9:1758. doi: 10.1038/s41598-018-36583-x (PMC6370840; doi:10.1038/s41598-018-36583-x)
Supplement: Supplementary file 1 — The effects of Co on the enhancement of magnetic properties by modifying the intergranular phase in Nd-Fe-B alloys [file 41598_2018_36583_MOESM1_ESM.pdf]

Supplementary Information: The effects of Co on the enhancement of magnetic properties by modifying the intergranular phase in Nd-Fe-B alloys

Y. Liang, Q. Deng, X. H. Tan, H. Li & H. Xu,

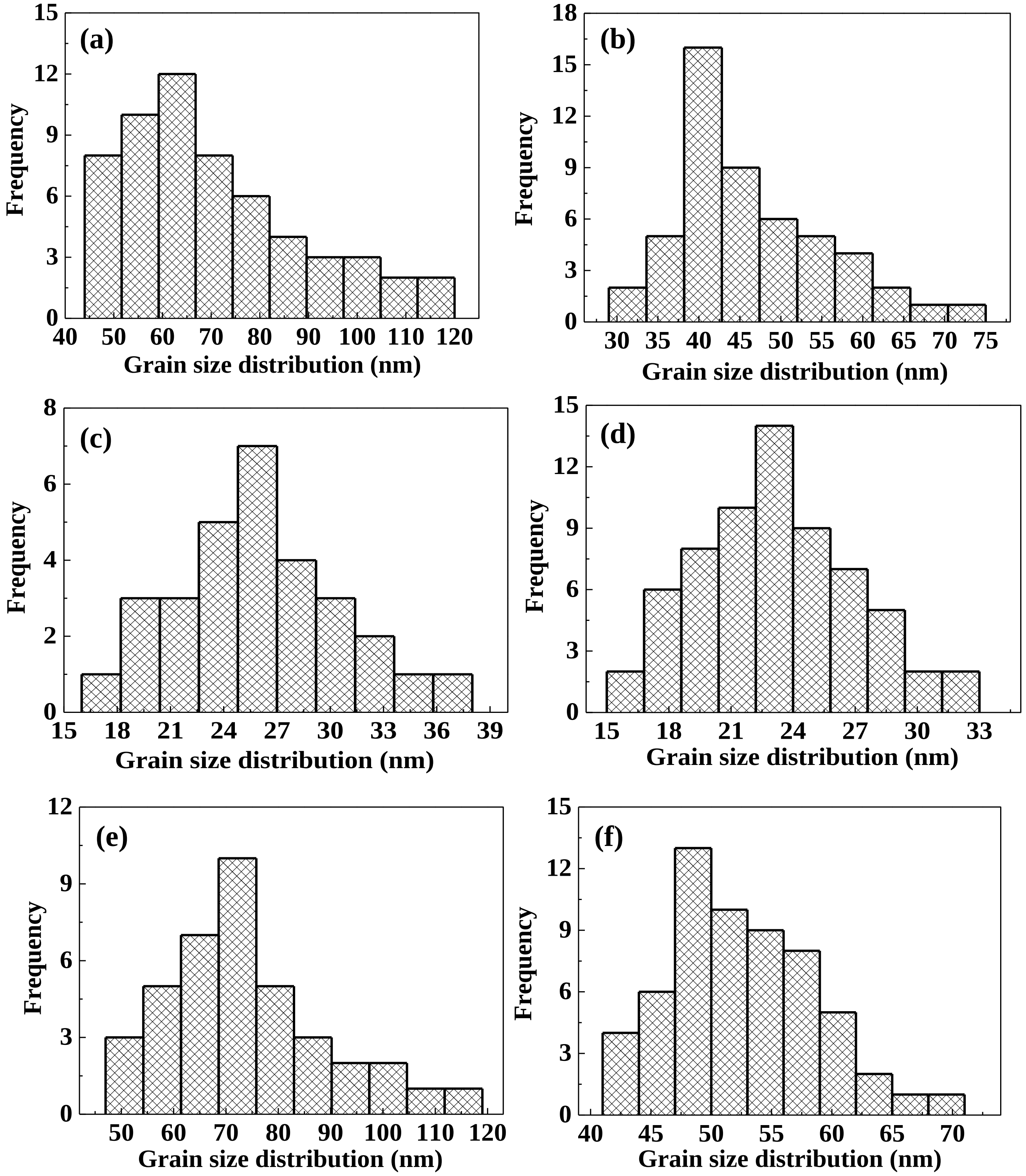

FIG. S1. Distribution histograms of grain size of the free surface (a, c, e) and the wheel surface (b, d, f) for $(Nd_{0.8}Pr_{0.2})_{2.2}Fe_{14-x}Co_xB$ (x=0, 2, 2.25) melt-spun ribbons.

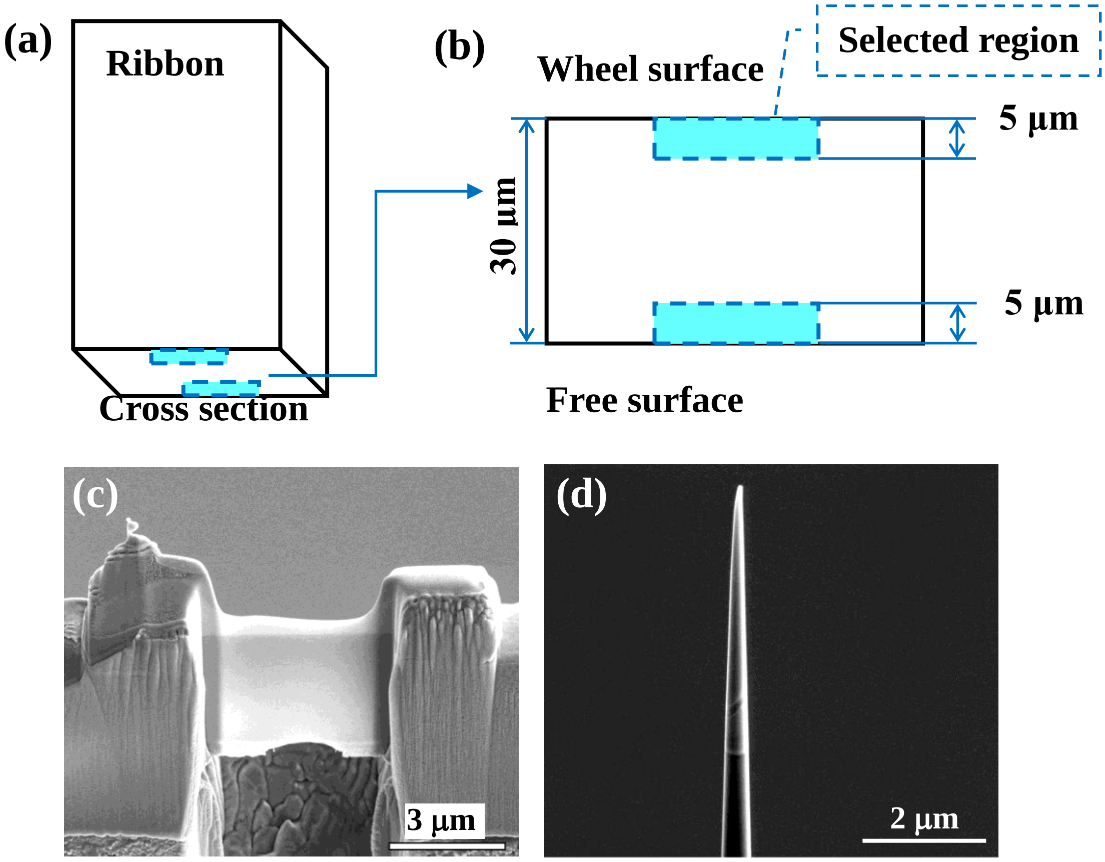

Figure S2. (a)(b) Selected regions for preparing cross-section samples near the wheel surface and free surface of ribbons; (c) TEM sample made by FIB; (d) APT needle sample made by FIB.
